# A multiply-add engine with monolithically integrated 3D memristor crossbar/CMOS hybrid circuit

**DOI:** 10.1038/srep42429

**Published:** 2017-02-14

**Authors:** B. Chakrabarti, M. A. Lastras-Montaño, G. Adam, M. Prezioso, B. Hoskins, K.-T. Cheng, D. B. Strukov

**Affiliations:** 1 Electrical and Computer Engineering Department, University of California, Santa Barbara, CA, 93106, USA; 2 Materials Department, University of California, Santa Barbara, CA, 93106, USA; 3 School of Engineering, Hong Kong University of Science and Technology, Clear Water Bay, Kowloon, Hong Kong

## Abstract

Silicon (Si) based complementary metal-oxide semiconductor (CMOS) technology has been the driving force of the information-technology revolution. However, scaling of CMOS technology as per Moore’s law has reached a serious bottleneck. Among the emerging technologies memristive devices can be promising for both memory as well as computing applications. Hybrid CMOS/memristor circuits with CMOL (CMOS + “Molecular”) architecture have been proposed to combine the extremely high density of the memristive devices with the robustness of CMOS technology, leading to terabit-scale memory and extremely efficient computing paradigm. In this work, we demonstrate a hybrid 3D CMOL circuit with 2 layers of memristive crossbars monolithically integrated on a pre-fabricated CMOS substrate. The integrated crossbars can be fully operated through the underlying CMOS circuitry. The memristive devices in both layers exhibit analog switching behavior with controlled tunability and stable multi-level operation. We perform dot-product operations with the 2D and 3D memristive crossbars to demonstrate the applicability of such 3D CMOL hybrid circuits as a multiply-add engine. To the best of our knowledge this is the first demonstration of a functional 3D CMOL hybrid circuit.

Resistance switching or memristive devices are metal-insulator-metal structures that can switch between at least two different resistance states upon application of an electrical impulse (voltage or current). Although the phenomenon of resistance switching has been known since the 1960 s, research interest has significantly grown in the last decade after resistance switching devices were identified not only as one of the leading candidates for next generation memory^1^,^2^,^3^,^4^,^5^,^6^,^7^ but also for analog computation^8^,^9^,^10^, neuromorphic circuits^11^,^12^,^13^,^14^,^15^, reconfigurable logic^16^,^17^ and other applications^18^,^19^. However, practical applications of memristive devices, which are passive circuit elements, may often need integration with active CMOS components. Recently a hybrid architecture termed as “CMOL” (CMOS + molecular devices) has been proposed that can combine the novel functionalities of memristive devices with CMOS technology to potentially lead to ultra-high density memory, reconfigurable logic and neuromorphic applications^20^,^21^,^22^. Performance of such hybrid circuits can be further improved manifold by integration of 3D memristive crossbars on CMOS technology. Reports of memristor-CMOS integration where memristors were fabricated between the two metal layers of a CMOS process exist in the literature^5^,^23^. H. Li *et al*. has recently shown 3D vertical resistive memory devices integrated with FinFET selectors^24^. The integrated devices were used to demonstrate in-memory computation capability. However, demonstrations of CMOL hybrid circuits has been extremely rare^16^,^25^. Xia *et al*. demonstrated reconfigurable logic functionalities with memristive components integrated on a CMOS substrate. However, only 2D planar devices were integrated. Moreover, discussions on the quality of interface between the CMOS and memristors as well as the memristor device characteristics were also very limited. We have been recently able to show 3D memristive crossbars with analog computation capability although the memristive crossbars were not integrated on a CMOS substrate^26^. In this work, we demonstrate vertical monolithic integration of 3D memristive crossbars on a foundry-processed 5 mm × 5 mm CMOS chip. High integration yield with low contact resistance between the CMOS layer and memristive devices was achieved. We demonstrate the dot-product operation, a critical computation for many applications involving linear transforms, using the 3D memristive crossbars with high-precision tunable analog devices. We believe that this work is a significant step towards practical realizations of highly efficient 3D hybrid CMOL circuits.

## Ultra-high bandwidth multiply-add engine with 3D CMOL hybrid circuits

**Figure 1:**
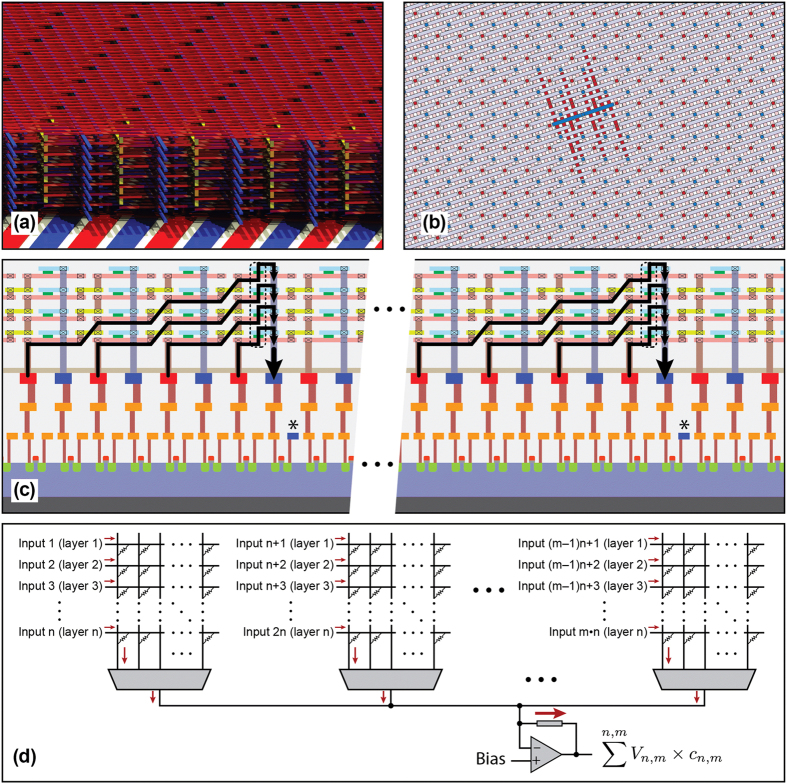
Conceptual representation of a 3D CMOL multiply-add engine. (**a**) Conceptual view of a 3D multi-layer crossbar integrated on CMOS substrate. The red and blue thinner wires represent the bottom and top electrodes of the crossbar respectively, the thicker red and blue wires illustrate the CMOS interconnections. (**b**) Top-view of one layer of a CMOL crossbar: the red and blue dots represent the contact vias for the bottom (BE) and top electrodes (TE) respectively. The highlighted electrodes (one TE and eight BE) demonstrate the CMOL connectivity. (**c**) Cross-sectional view of (**b**) showing multiply-add operation at the ‘blue’ pin of the eight input signals fed through the eight ‘red’ pins. (**d**) Multiply-add operation in a (m × m × n) 3D crossbar where n is the number of layers.

Figure 1 illustrates a high bandwidth multiply-add engine using a 3D CMOL hybrid circuit. A memristive crossbar array can naturally perform dot-product operation. If an array of voltage signals is applied to the rows of a crossbar, the current measured at a column will be a weighted summation of the inputs with each input signal being multiplied by the conductance or ‘weight’ of the corresponding cross-point memristive device. Multiply-add operation is performed concurrently in all the layers as well within each layer of crossbar and the resulting currents are summed at the output using the CMOS circuitry. Such massive parallelism and the effectiveness of CMOL area-distributed interface enable very high bandwidth computations, which can be further improved in 3D CMOL circuits^27^. Figure 1a is an illustration of a 3D CMOL crossbar with 8 layers, while Fig. 1b shows one layer of a CMOL crossbar. The red and blue via openings provide connectivity to the area-distributed interface of the CMOS substrate for the BE and TE arrays of the memristive crossbar. Each TE (blue line) in this CMOL architecture is connected to eight BE (red line). Figure 1c is a partial cross-sectional view of the 3D CMOL in Fig. 1a showing only 4 layers. In each layer 8 BE rows are connected to a TE column through 8 memristive devices while the TE columns are connected to one another through 8 vertically stacked devices. The overall multiply-add operation therefore has 2-dimensional and 3-dimensional components. Within one layer each TE line receives the weighted input signals from eight BE lines, each weight corresponding to the conductance state of the individual memristors. The output signals generated at each TE line in each layer are then summed at the TE column over all the layers. Figure 1d shows the equivalent circuit representation of the dot product operation in the 3D crossbar.

In the next sections we will discuss practical implementation of a 3D CMOL multiply-add engine. For demonstration purposes our 3D CMOL hybrid circuit consists of 2 layers of memristive crossbars. However, this process can be extended to fabricate as many crossbar layers as needed.

## Results

### 3D Crossbar integration on CMOS substrate

**Figure 2:**
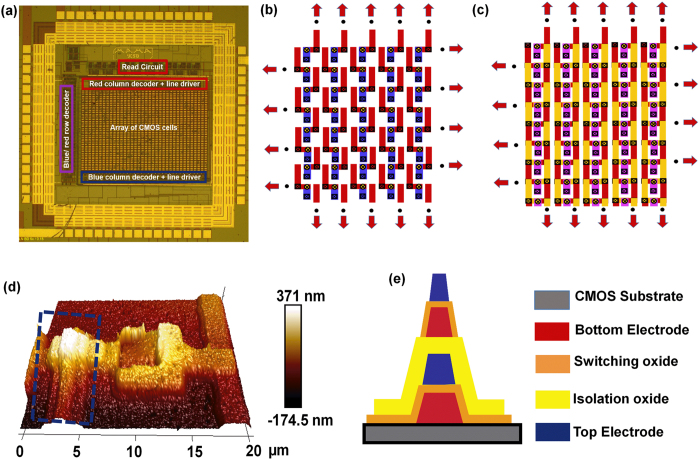
Fabrication of a 3D CMOL crossbar. (**a**) Optical image of the foundry-processed CMOS chip showing the on-chip decoder, ‘Read’ and ‘Write’ circuitry as well as the 24 × 36 array of CMOS cells. Each ‘CMOS cell’ houses a pair of pins (‘Red’ and ‘Blue’), (**b**) structure of the first layer of the 3D crossbar, (**c**) the final structure of the 3D crossbar with 2 layers (**d**) AFM image of a section of the 3D crossbar: the highlighted section shows two stacked device layers, (**e**) Cross-section of the region highlighted in (**d**).

Figure 2a shows an optical image of a typical CMOS chip designed for fabrication of the hybrid circuits. The chip, a 5 mm × 5 mm die was fabricated in a commercial foundry with 0.5 μm technology. The area-distributed interface required for the CMOL architecture consists of an array of ‘CMOS cells’ in the top metal level (Al) with each cell containing a pair of pads (Red and Blue). The chip also houses the decoder, ‘Read’ and ‘Write’ circuits for addressing as well as writing and sensing operations on the integrated memristive crossbars. More details of the chip level architecture and the ‘Read’ and ‘Write’ circuitry on the CMOS chip is discussed in the next section. A scratch-protect oxynitride layer covers the CMOS pads. The topography on the top surface (see Supplementary Figure 1a,b) originates from the thickness of the Al pads in the top metal layer. The severe topography over the area-distributed interface (>800 nm) necessitates planarization before memristive crossbars can be fabricated on top. Among the different planarization techniques available only chemical mechanical planarization (CMP) provides both local and global planarity. However, CMP is suitable for wafer level applications and reports of CMP on die level are extremely rare. In one demonstration approximately 5 × 5 mm^2^ chips were planarized using multiple 10 × 10 mm^2^ dummy Si pieces around the chip^28^. However this method is unlikely to produce reproducible results as the relative height distribution between the dummies and the actual sample will vary between experiments. To achieve reproducible planarization we use a 4 inch Si wafer with a cavity slightly larger than the die. The thickness of this wafer (holder) was carefully adjusted so that the holder is approximately 3–4 μm thinner than the CMOS chip (see methods and Supplementary Figure 2 for details). The scratch-protect oxynitride on the CMOS chip was removed and 2.5 μm silicon dioxide (SiO_2_) was deposited before polishing (see methods and Supplementary Figure 3). Both the holder and the chip were then attached on to a second Si carrier wafer by crystal bond (Supplementary Figures 2 and 3) and the whole assembly was polished by CMP. Planarization was performed in two steps. The first step employs a fast polishing that reduces the topography from >800 nm to ~30–40 nm (Supplementary Figure 4b,c). In the second step the topography is further reduced by a slow polish down to <10 nm (See Supplementary Figure 4d,e). The surface roughness after planarization process can be a key factor for reliable device performance. In our experiments, the post-CMP surface roughness (<4 nm, see Supplementary Figure 6) is negligible compared to the thickness of the switching oxide (~33 nm) and creation of false devices due to roughness can be ruled out. The memristive crossbars were fabricated on top after the planarization (see methods and Supplementary Figure 7 for details). Note that in this experiment we performed the planarization process once to remove the initial topography originating from the CMOS top-metal layer since we fabricated only 2 layers for demonstration. However, for integration of more than 2 layers where each layer would add further topography additional planarizations after fabrication of each crossbar layer would be required.

Figure 2b,c show the structure of the integrated 3D crossbar. Figure 2b shows a section of the first layer of the crossbar with the arrows indicating continuation of the structure in the indicated directions. Here the red and blue lines indicate the BE and TE of layer 1. Each electrode (BE or TE) is connected to an underlying CMOS cell through a BE or a TE via. For the BEs, the via is through the planarized SiO_2_ layer while for the TE the via opening is through the switching oxide+ the SiO_2_ layer (see methods for fabrication details). The BEs of the 2^nd^ layer (yellow lines) are connected to the BEs of the 1^st^ layer by a set of vias through the isolation oxide (SiO_2_) between the 1^st^ and the 2^nd^ layer. The TEs of the 2^nd^ layer are fabricated on top of the TEs of the 1^st^ layer. As such, this structure allows integration of more than 2 layers without any additional lithography masks. For example a 3^rd^ crossbar layer will have the BEs in the same positions as in the BEs of the 1^st^ layer (Fig. 2c) while the TEs of the 3^rd^ layer can be fabricated on top of the TEs of the 1^st^ and 2^nd^ layers. For test purposes we also fabricated single devices and 2-dimensional crossbars (See Supplementary Figure 8). The devices (both for 2D and 3D crossbars) employ a bilayer Al_2_O_3_/TiO_2-x_ dielectric as the switching material. The non-stoichiometry of the TiO_2-x_ layer is controlled by the sputtering conditions. The devices have Ta/Pt BEs and Ti/Pt TEs.

### CMOS circuit details

**Figure 3:**
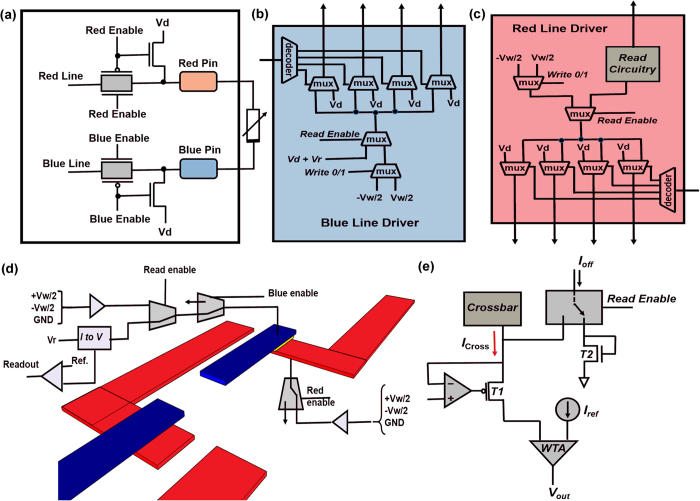
CMOS circuitry. (**a**) Circuit of a CMOS cell. (**b**,**c**) Blue and Red line driver for supplying the appropriate biases to the Blue and Red pins respectively. (**d**) Cartoon of the write/read operation for the memristive crossbars. (**e**) Schematic of the sensing circuit.

As mentioned before, addressing the memristive crossbars as well as the memory operations are all performed through built-in circuitry in the CMOS chip itself. Figure 3a shows the circuit details of a CMOS cell which contains two transmission gates that connect the red and blue pins to the red and blue lines respectively when the gates are enabled. The red and blue lines provide the appropriate read/write voltages to the pins. Both the red and blue pins are connected to a default voltage *V*_d_ when the transmission gates are disabled. The red and blue drivers shown in Fig. 3b,c supply the required voltages to the red and blue lines. Depending on the memory operation (read/write) the blue and red drivers can supply a read voltage of *V*_r_ and write voltage of ±*V*_w_/2 in both the drivers. Row/column decoders are employed to select the appropriate ‘Blue’ and ‘Red’ pins for applying ‘read’/’write’ bias to the desired crosspoint (s). The row and column decoders on the chip surround the array of CMOS cells (see Fig. 2a). The entire CMOS cell array is divided into many units called multi-cells. A unique crosspoint within one multi-cell can be chosen using a 12 bit address provided as user input through the interface we have developed. At a time eight such crosspoints corresponding to eight multi-cell columns can be selected concurrently, leading to eight bit operation (Supplementary Figure 9). More details on the addressing scheme for the memristive crossbars can also be found elsewhere^29^. Figure 3d shows a cartoon of the ‘Read’/‘Write’ operations on the integrated crossbars. For simplicity only the first layer of the crossbar structure is shown (see also Fig. 2b). During the ‘Write’ operation a bias of either +*V*_w_/2 (set) or −*V*_w_/2 (reset) is applied through the blue line driver to the TE (blue wire), while the BE is connected to bias of −*V*_w_/2 (set) or (+*V*_w_/2). This leads to application of a resultant bias of +*V*_w_ (set) or −*V*_w_ (reset) across the device. During a ‘Read’ operation a bias of +*V*_r_ is applied to the TE (with respect to the BE). A schematic of the ‘Read’ circuitry is shown in Fig. 3e. When the Read Enable signal is asserted, the current from the crossbar *I*_Cross_ is sensed through a winner-take-all (WTA) circuit by comparing against a reference current *I*_ref_. When the Read Enable signal is not asserted, a very small current *I*_off_ (50 pA) is passed through the WTA circuitry to avoid a delay in the loop for the Read operation^30^.

### Switching characteristics in 2D and 3D devices

**Figure 4:**
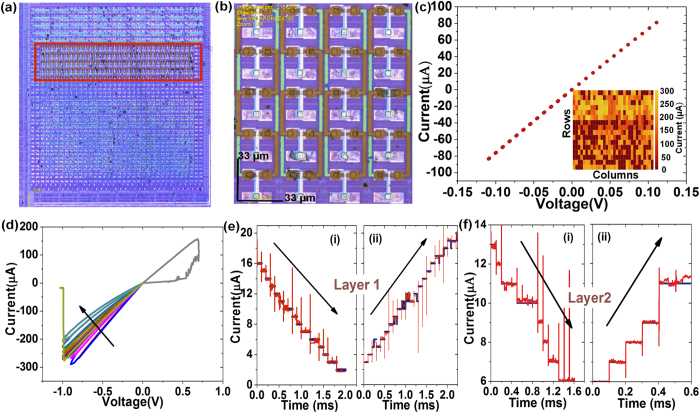
Electrical characteristics. (**a**) An optical image of the CMOS chip with vertically integrated memristive crossbars: the region highlighted in the red box has the 3D CMOL crossbar structures, (**b**) A high-resolution optical image of a section of the integrated 3D crossbars. (**c**) A typical *I-V* plot measured on a test structure to test the contact resistance between the memristive layer and the underlying CMOS. Inset shows a map of the current measured at all the 24 × 36 array of ‘red pins’ showing contact between the integrated devices and the CMOS cells. Current is measured at 0.2 V for each measurement. (**d**) An I-V plot showing typical DC switching characteristics of the integrated memristive devices: the device is initially turned on and is gradually reset demonstrating analog switching behavior, (**e**) pulsed switching characteristic of a device in layer 1 showing gradual reset and set operation: the device is tuned to each desired state using a tuning algorithm (discussed in the supporting info.). The arrow indicates the direction of programming in each case, (**f**) pulsed switching characteristic of a device in layer 2 showing gradual reset and set operation: the device is tuned to each desired state using the tuning algorithm.

Figure 4a shows an optical image of the processed CMOS chip. The highlighted region contains the integrated 3D crossbars. Integrated 2D crossbars and single devices can be seen in other regions on the area-distributed interface. A zoomed-in view showing a section of the integrated 3D crossbars can be seen in Fig. 4b. The underlying CMOS pads can be clearly seen as well. Figure 4c shows *I-V* characteristic of a test structure to evaluate contact resistance. The test structure has the BE and TE shorted with no switching dielectric in between. The measured resistance for the specific test structure is ~1.5 kΩ. Figure 4c inset shows a map of the measured current at all the contacts between the CMOS pads and the bottom electrodes (‘red’ pins) of the 3D crossbars. More than 90% of the contacts (145 out of 160) have current of 100 μA or more at a V_read_ of 0.3 V indicating that most of the contacts between the memristive devices and the underlying CMOS substrate have contact resistance less than 3 kΩ. This is an important step towards realization of large-area CMOL circuits. Figure 4d shows typical DC switching characteristics of an integrated memristive device. The devices are initially in a low resistance state. Analog reset operation is observed as the reset-stop voltage is increased incrementally. A more abrupt reset operation can be achieved with sufficiently high reset bias (~−1 V). Similarly the devices can be turned on significantly with a positive bias ~0.8 V. No current compliance was used to control the set operation. The device can be turned more on to a less resistive state by applying a higher set bias. However, an internal compliance (~250 μA) employed by the CMOS circuitry protects the devices from permanent damage. This internal compliance is tunable through the CMOS circuitry. The switching bias is always applied at the TE while the BE is connected to electrical ground. It is to be noted that abrupt change in device conductance can occur, especially under stress at voltages similar to the maximum voltage applied for analog switching. For example the abrupt reset in Fig. 4d occurs after several consecutive analog reset steps. However, any abrupt change in the conductance is adjusted and the device is tuned to the desired state using a write-and-verify tuning algorithm. The algorithm employs pulse trains of increasing amplitude to incrementally increase/decrease the resistance of the devices with controllable precision. Figure 4e, f exhibit examples of high-precision multi-level tuning operation for the 1^st^ and 2^nd^ layer devices in a 3D crossbar, respectively (See Supplementary Figure 10 and Supplementary note 1 for a detailed description of the tuning algorithm). The tuning operation was optimized to minimize the number of iterations required to tune to each state. The number of pulses required to tune the device to each level was used as a measure of the tuning speed. Optimization was achieved by adjusting the user-defined inputs of the tuning procedure (Supplementary Figures 10 and 11). An optimized tuning algorithm with 10% precision was employed to tune the devices shown in Fig. 4e and f. The red lines in each figure indicate the actual current during the tuning operation while the blue lines indicate the desired states. Devices in both layers can be tuned to at least 8 levels with 10% precision (see also Supplementary Figure 12). Under the optimized conditions all the levels can be tuned within 150 applied pulses (Supplementary Figure 12). In a typical experiment we also tuned the devices to 8 distinct levels with each level being programmed 1000 times. All the levels exhibit clearly distinguishable distribution over 1000 switching operations showing stable endurance under the tuning procedure (Supplementary Figure 12). Stable room temperature retention was observed as well up to 10^4^ seconds (Supplementary Figure 12). Note that the pulses applied in the tuning procedure have amplitudes significantly lower than the amplitudes required to turn the devices fully on/off (Supplementary Figure 13). As resistive switching devices typically have strongly non-linear switching dynamics with respect to applied voltage^31^, it is expected that the stress caused by the tuning procedure is significantly less compared to the switching of the devices fully on/off.

### Dot product operations with 3D crossbar devices

**Figure 5:**
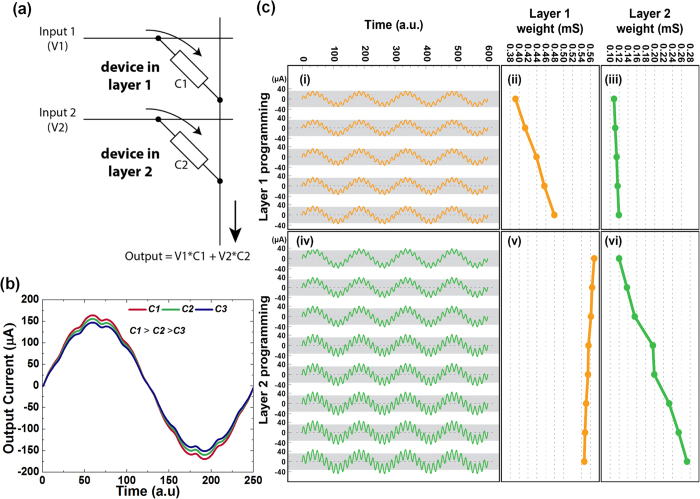
Dot-product operation in the integrated 3D memristive crossbars. (**a**) Schematic of the set-up for the dot-product operation utilizing two devices in two different layers of the 3D crossbar, (**b**) An example of the dot product operation with two sinusoidal inputs applied to two devices in a 3D crossbar and the device in layer 1 being programmed to decreasing conductance values, (**c**) (i) evolution of the output waveform with the weight of the device in layer 1 changing (ii) and the weight for the device on the 2nd layer being kept constant (iii); (iv) evolution of the output waveform when weight of the device in layer 1 is kept constant (v) while layer 2 device changes it’s state (weight) (vi).

Figure 5 describes the multiply-add operation utilizing the 3D memristive crossbars. We would like to emphasize that the entire multiply-add operation is carried out through the in-built CMOS circuitry in accordance to the write and sensing schemes discussed previously. An entire 3D crossbar with 5 devices in each layer was employed for the operation. However, for the simplicity of demonstration we only programmed one device in each layer during the operation. Figure 5a represents the set-up used for the dot-product operation. Input voltage signal V1 is applied to the TE (Blue pin) of the layer 1 device (conductance C1) while signal V2 is applied to the TE of the device in layer 2 (conductance C2). The summation of the current outputs of the devices is carried out at the two connected BE (red) terminals. The output is a weighted summation of the input voltage signals. Figure 5b shows an example of the multiply-add operation with the bottom layer device being programmed to different states. The input signals for both bottom and top layer devices have an amplitude of 300 mV. However, the frequency of the signal for channel 2 is 10x more than that of channel 1. The evolution of the output current waveform shown in Fig. 5b reflects the multiply-add operation as the conductance of the 1^st^ layer device (channel 1) varies from C1 to C2 to C3 in the decreasing order. With the reduction of weight for device 1 the amplitude of the low frequency component (envelope) of the output waveform reduces while the amplitude of the high-frequency component (ripples on the envelope) remains unchanged. Figure 5c provides more examples of the dot-product operations in another set of 3D memristive crossbars. In the experiment shown in Fig. 5c we first change the weight of the layer 1 device while keeping the state of the layer 2 device unchanged. Figure 5c(ii) shows a gradual increase of channel 1 (layer 1) weight from 0.39 mS to 0.48 mS in 5 steps, using the tuning algorithm. The evolution of the output waveform is shown in Fig. 5c(i), which confirms the correct operation. The amplitude (peak-peak) of the output current for the layer 1 component changes from 72 μA to 95 μA as a result (see Supplementary Figure 14b). In the next step, the state of the device in layer 1 is kept unchanged while the device in layer 2 is gradually turned on (Fig. 5c(v)). The corresponding output waveforms are displayed in Fig. 5c(iv). As expected, as the weight for channel 2 increases the amplitude of the high-frequency component in the output waveform increases. As shown in Supplementary Figure 14f, the layer 2 component (peak-peak) changes from 10–50 μA. The margins for the change in output current components for both the layer 1 and layer 2 devices are ~5 μA or more (Supplementary Figure 14) and it is to be noted that this margin can be easily adjusted by the tuning procedure. These results indicate that the devices in each layer in the 3D CMOL crossbars can be controlled independently and used for matrix multiplication operation. However, practical implementation of high bandwidth multiply-add operation using 3D CMOS/memristor crossbars will also require overcoming the challenges due to finite line resistance, sneak-path and other sources of noise. Increase in the number of layers in a 3D crossbar is equivalent to increasing the size of a 2D crossbar array, thereby also increasing the sneak-currents^32^. Several remedies proposed to mitigate the effect of sneak currents include use of mapping algorithms (to map target weight matrix onto actual crossbar conductance values)^33^ or train the hardware through supervised or unsupervised learning schemes^14^,^34^. A detailed analysis of the effect of sneak-paths in 3D hybrid memristor/CMOS and accurate benchmarking is beyond the scope of this work. Development of a selector technology can also be a possible solution. We will investigate these avenues in our future work.

In summary, we demonstrate the first 3D CMOL hybrid circuit with 3D memristive crossbars monolithically integrated on a CMOS substrate. High integration yield in terms of good electrical contact between the memristive components and the CMOS substrate was achieved by planarization of the CMOS chips. The integrated 3D crossbars can be fully controlled by the underlying CMOS circuitry. The memristive devices display forming-free switching with low voltage operation. They are analog tunable using a write-and-verify algorithm. The multi-level control of the states for the memristive devices allows them to be used in multiply-add operations where their conductance values can be used as controllable weights. Demonstration of multiply-add operation utilizing memristive devices both in the 1^st^ and 2^nd^ layer of the 3D crossbars opens up promise for ultra-high bandwidth multiply-add engines with high density memristor/CMOS 3D hybrid circuits.

## Methods

### Preparation of planarization holder

The CMOS chips used in this experiment have a dimension of 5 mm × 5 mm and a thickness of 256 μm. Thickness of a 4 inch Si wafer with initial thickness of ~260 μm (+0−4 μm) is reduced by 6–10 μm (depending on the initial thickness) using deep-Si Reactive Ion Etching (DRIE) to have a final thickness of ~254 μm. The wafer is then polished by CMP in SF1 slurry (alkaline colloidal silica) for 4 mins to remove the roughness generated by the DRIE process. A 3 μm SiO_2_ film is then deposited by Plasma Enhanced Chemical Vapor Deposition (PECVD) on the wafer. A 5.5 mm window is patterned on the oxide by photolithography with negative resist (AZnLOF2020) and using a 5.5 mm Si piece as the mask. The oxide in the window region is etched back with CHF3 plasma. The wafer is then subjected to DRIE to completely etch Si within the window to make a 5.5 mm × 5.5 mm hole in the Si wafer.

### Chemical mechanical planarization of the chip

The as-received chip has a 1.3 μm scratch-protect oxynitride layer with unknown composition (undisclosed from the Foundry). Due to the unknown composition/quality of the oxynitride it is difficult to precisely control processing of this layer. Therefore we completely remove this layer and use a planarization dielectric of known quality/composition. After completing removing the oxynitride layer by dry etching in CHF_3_ plasma, the organic residues were removed by cleaning in AZ300T for 15 minutes. Next the CMOS pads in the active region are covered with Ti/Au (10/100 nm) to prevent oxidation of Al. A 2.5 μm SiO_2_ layer is then deposited by Inductively Coupled Plasma based PECVD (ICP-PECVD) at low temperature (50 °C). The planarization holder is then crystal bonded on a second Si substrate and the CMOS chip is placed in the middle of the holder. The entire ensemble was then polished in CMP with SF1 slurry for 4 mins. After CMP the final topography of the chip is verified by atomic force microscopy (AFM). The oxide thickness on top of the CMOS pads post-CMP is measured by a reflectance measurement unit. The post-CMP oxide thickness is ~1.5 μm across the chip. Next the planarization oxide (SiO_2_) is etched back to the desired thickness (180 nm).

### 3D memristive crossbar fabrication

4 × 4 μm^2^ via holes for contact between the BEs of the crossbars and the CMOS pads are first created by photolithography and CHF_3_ plasma etching through the 180 nm planarization oxide. Next, Ta/Pt (5/60 nm) BEs for layer 1 devices (width 2 μm) were patterned by photolithography and E-beam evaporation. The Al_2_O_3_/TiO_x_ (3/30 nm) switching stack is deposited by reactive sputtering in Ar/O_2_ plasma. Stoichiometry of the TiO_x_ layer was controlled by controlling the O_2_ flow. Next, via holes (4 × 4 μm^2^) for contact between the TEs and CMOS pads are created by photolithography and dry etching in CHF_3_ plasma. TEs of Ti/Pt (15/60 nm) are defined by optical lithography and E-beam evaporation. After fabrication of the first crossbar layer an isolation oxide of 200 nm is deposited by ICP- PECVD. The 2^nd^ layer of crossbars is fabricated by performing the same fabrication steps used for layer 1, namely patterning of BE via holes, deposition of Ta/Pt BEs, deposition of the switching oxide stack, patterning of TE via holes and defining the Ti/Pt TEs. In a final lithography step, via holes are opened on the wire-bonding pads by photolithography and CHF_3_ plasma etching. The chip is then annealed at 300 °C for 15 mins in forming gas (N_2_ + H_2_). The processed chip was wire bonded and packaged in a commercial facility before electrical measurements were performed.

## Additional Information

**How to cite this article**: Chakrabarti, B. *et al*. A multiply-add engine with monolithically integrated 3D memristor crossbar/CMOS hybrid circuit. *Sci. Rep.* **7**, 42429; doi: 10.1038/srep42429 (2017).

**Publisher's note:** Springer Nature remains neutral with regard to jurisdictional claims in published maps and institutional affiliations.

## Supplementary Material

Supplementary Information
