# Corrigendum: A multiply-add engine with monolithically integrated 3D memristor crossbar/CMOS hybrid circuit

**DOI:** 10.1038/srep46874

**Published:** 2017-07-27

**Authors:** B. Chakrabarti, M. A. Lastras-Montaño, G. Adam, M. Prezioso, B. Hoskins, M. Payvand, A. Madhavan, A. Ghofrani, L. Theogarajan, K.-T. Cheng, D. B. Strukov

Scientific Reports 7: Article number: 42429; 10.1038/srep42429 published online: 02 14 2017; updated: 07 27 2017.

M. Payvand, A. Madhavan, A. Ghofrani and L. Theogarajan were omitted from the author list in the original version of this Article. This has been corrected in the PDF and HTML versions of the Article, as well as in the Supplementary Information that now accompanies the Article.

The Author Contributions section now reads:

B. C. wrote the manuscript and fabricated the hybrid CMOS/3D memristor chip. M. A. L.-M. and A. G. designed the architecture and the digital circuitry of the CMOS chip. M. P. (Payvand) designed the analog circuitry and M. P. (Payvand) and A. M. designed the overall layout of the CMOS chip. M. A. L.-M. created the user-interface for electrical measurements. Both B. C. and M. A. L.-M. conducted the electrical characterizations and analyzed the data. G. A. contributed in the chemical mechanical planarization of the CMOS chip as well as the tuning operation of the memristors. M. P. (Prezioso) contributed to develop strategies for electrical characterization of the memristor crossbars. B. H. was involved in the non-stoichiometric TiO2-x thin film depositions. L. T., K. T. C. and D. B. S. have supervised the overall project. All authors have seen and approved of the manuscript before submission.

