# Supplementary material for: A multiply-add engine with monolithically integrated 3D memristor crossbar/CMOS hybrid circuit
Source: Sci Rep. 2017 Feb 14;7:42429. doi: 10.1038/srep42429 (PMC5307953; doi:10.1038/srep42429)
Supplement: Supplementary Information [file srep42429-s1.pdf]

A multiply-add engine with monolithically integrated 3D memristor crossbar/CMOS hybrid circuit

B Chakrabarti[1*], M A Lastras-Montaño[1], G Adam[1], M Prezioso[1], B. Hoskins[2], K-T Cheng[1,3] and D B Strukov[1]

[1] Electrical and Computer Engineering Department, University of California, Santa Barbara, CA, 93106.

[2] Materials Department, University of California, Santa Barbara, CA, 93107.

[3]School of Engineering, Hong Kong University of Science and Technology, Clear Water Bay, Kowloon, Hong Kong.

E-mail: bchakrabarti@ece.ucsb.edu

Supplementary Information

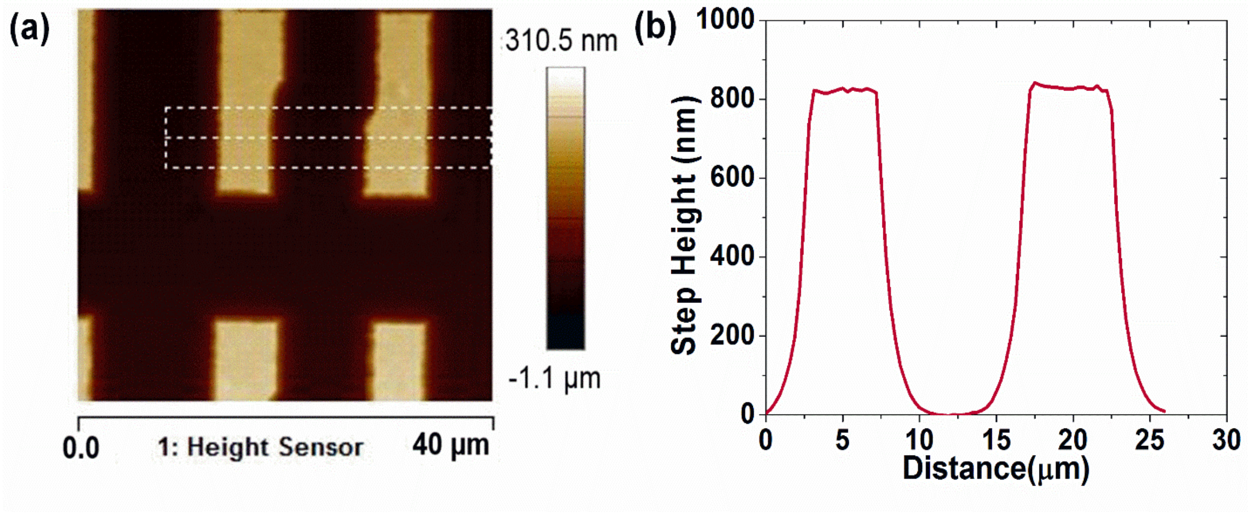

Supplementary figure 1. Initial topography (before planarization) of the as-received CMOS chip. (a) AFM image of a section of the CMOS chip before planarization showing the CMOS pads covered under SiO_2, **(b)** the topography of the region shown in (a).

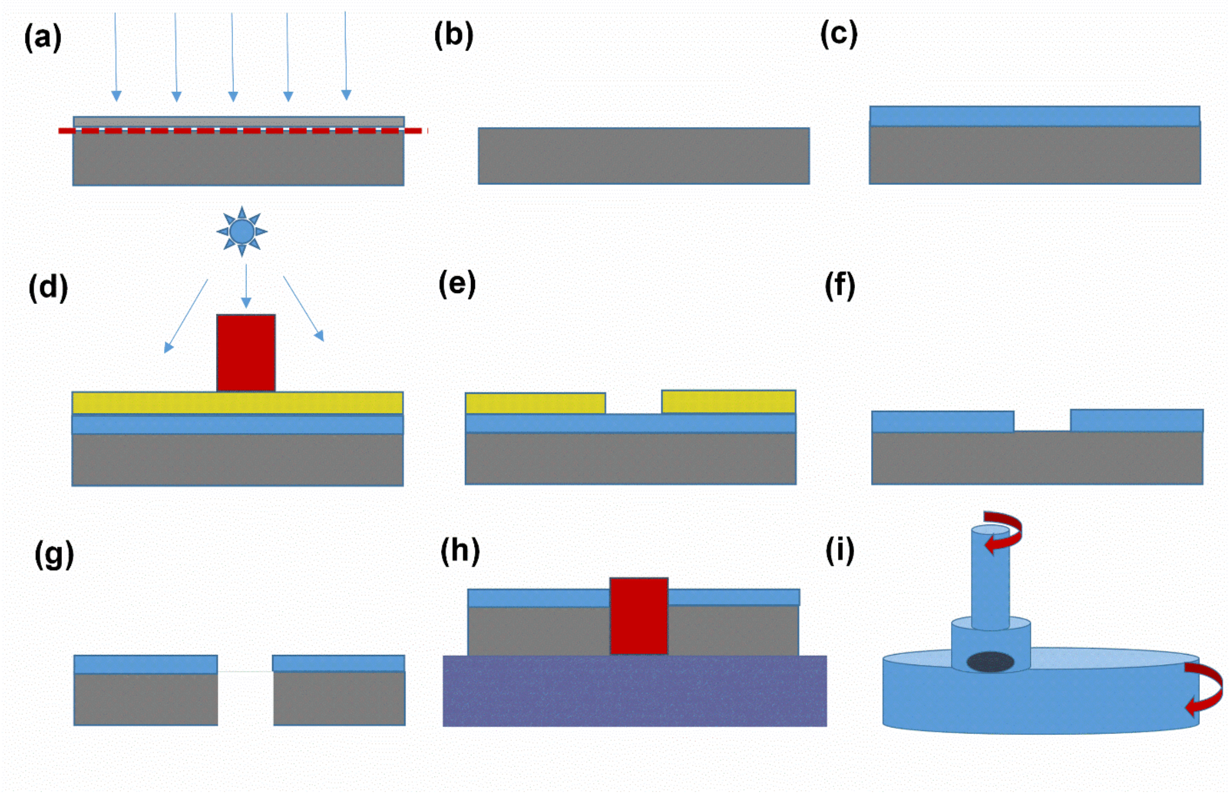

Supplementary figure 2. Preparation of the CMP holder followed by the planarization process. **(a)** initial deep-Si Reactive Ion Etching (DRIE) and planarization by CMP on the as received wafer (4 inch) to reduce the wafer thickness to desired value, **(b)** holder wafer after thickness reduction by CMP, **(c)** 3 µm oxide deposition by PECVD, **(d)** photolithography to define the etch window through the oxide using a dummy chip (6 mm), **(e)** profile of the resist after lithography and development, **(f)** dry etching and removal of oxide from the etch window, **(g)** etch through the Silicon wafer at the etch window by deep-Silicon Reactive Ion Etching (DRIE) process to make a cavity in the Silicon wafer, **(h)** a chip is placed inside the cavity of the holder and the whole assembly is bonded onto another 4 inch Silicon wafer by crystal bond: note that the height of the chip is slightly higher than the cavity (~ 3-4 µm) to ensure that during the CMP process the chip will be planarized, **(i)** the chip-holder-carrier assembly is planarized in the CMP tool.

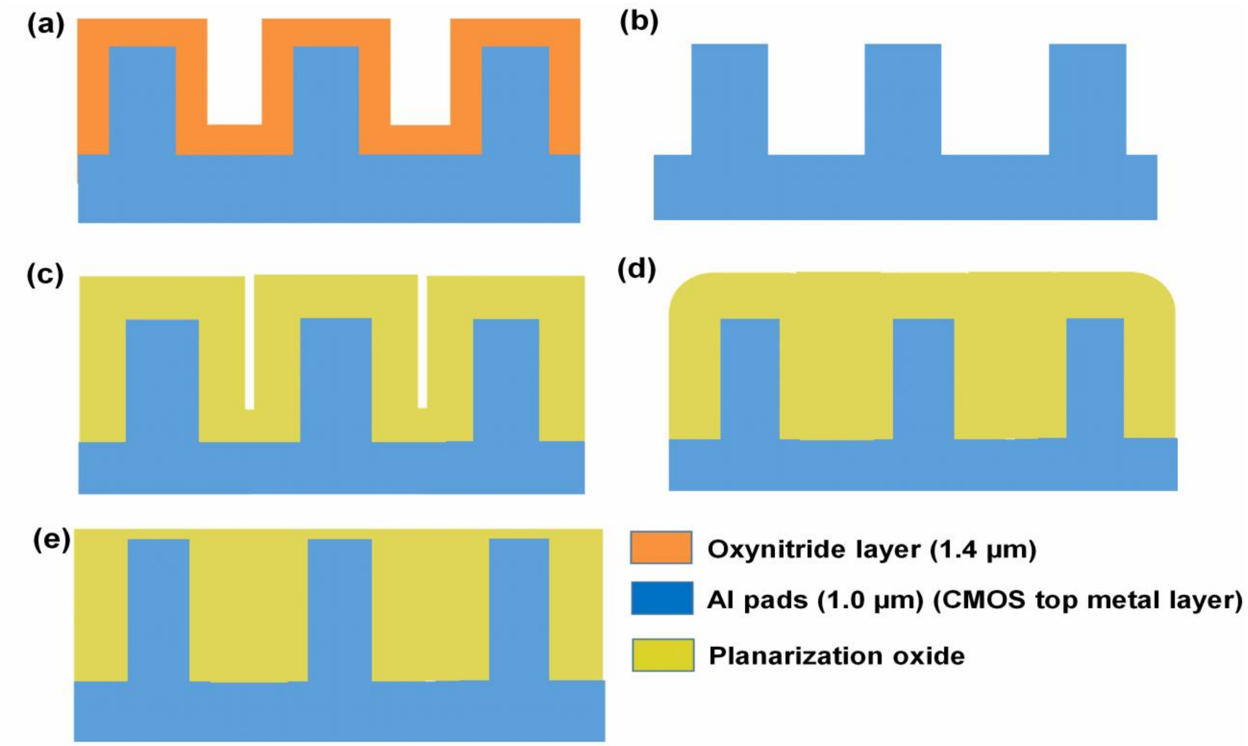

Supplementary figure 3. Planarization and etch back procedure. (a) The as-received chip with the scratch protect oxynitride covering the top metal (Al) of the CMOS layer, **(b)** removal of the oxynitride layer by plasma etching, **(c)** deposition of silicon dioxide by low temperature plasma enhanced chemical vapor deposition (PECVD): thickness of the oxide deposited is twice that of the thickness of the Al pads of the CMOS layer, **(d)** planarization of the oxide by chemical mechanical planarization (CMP) process, **(e)** etch-back of the planarization oxide after CMP by plasma etching to a desired thickness (~180 nm).

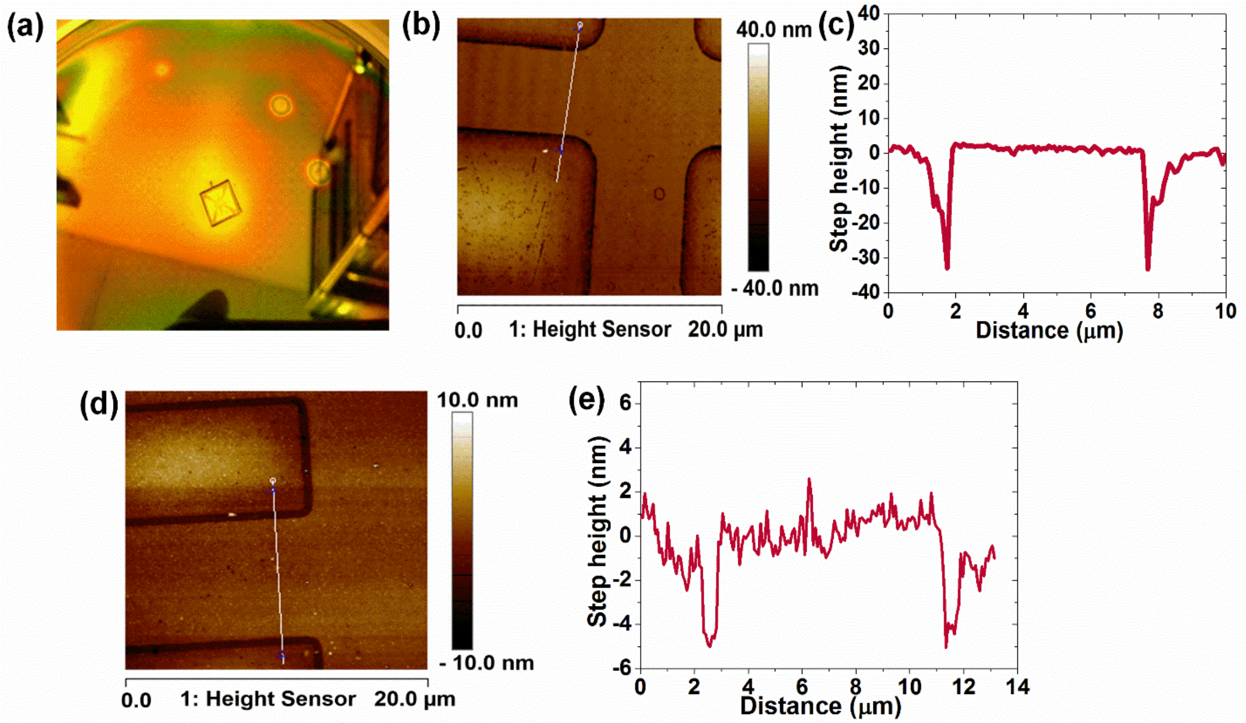

Supplementary figure 4. Surface topography after planarization. (a) Optical image of a CMOS chip inside the cavity of a Silicon carrier wafer with both the carrier and the chip crystal-bonded on to another Silicon holder wafer, **(b)** AFM image of a section of the CMOS chip after the first planarization step (fast polish) showing the CMOS pads covered under SiO_2. **(c)** The topography of the region shown in (b) indicates step-height reduction down to ~ 30-40 nm. **(d)** AFM image of a region after the final planarization (slow polish). **(e)** Topography of the region shows step height less than 10 nm.

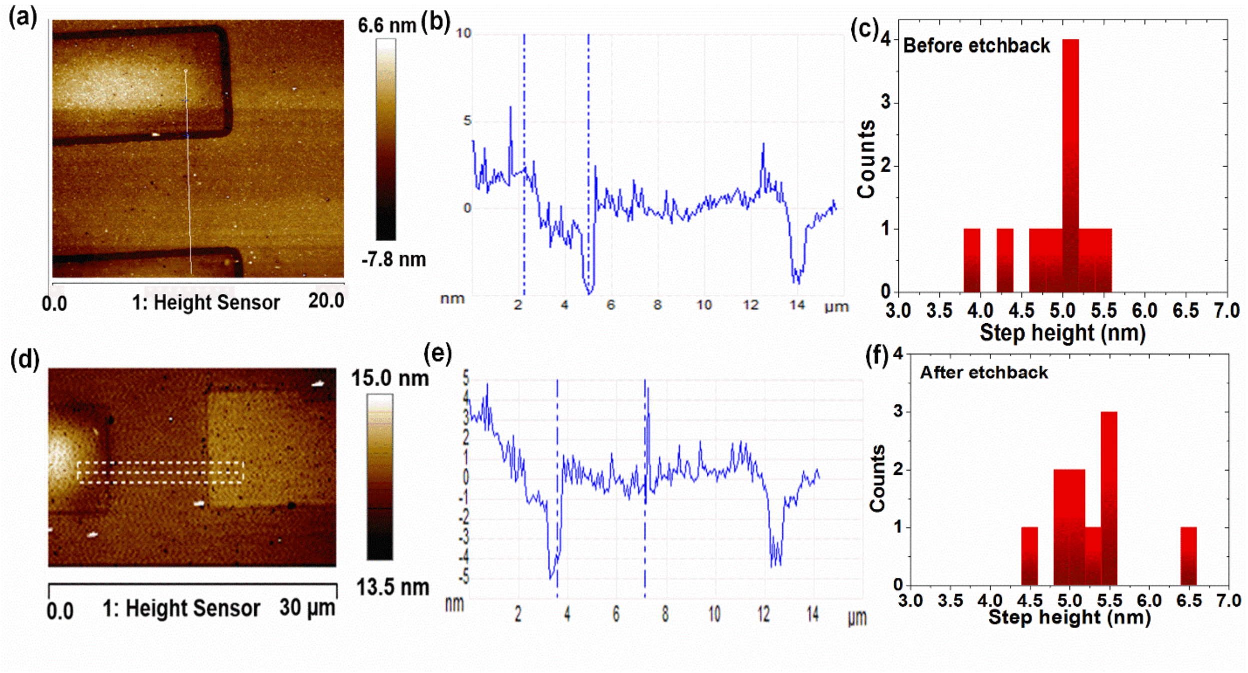

Supplementary figure 5. Step height measurements before and after the etch-back procedure. (a) AFM image of CMOS pads under planarization oxide before the etchback process, **(b)** surface topography of the region shown in (a), (c) histogram showing distribution of surface roughness in the region, **(d)-(f)** AFM image, surface topography and histogram of step height on the planarization oxide after the etchback process.

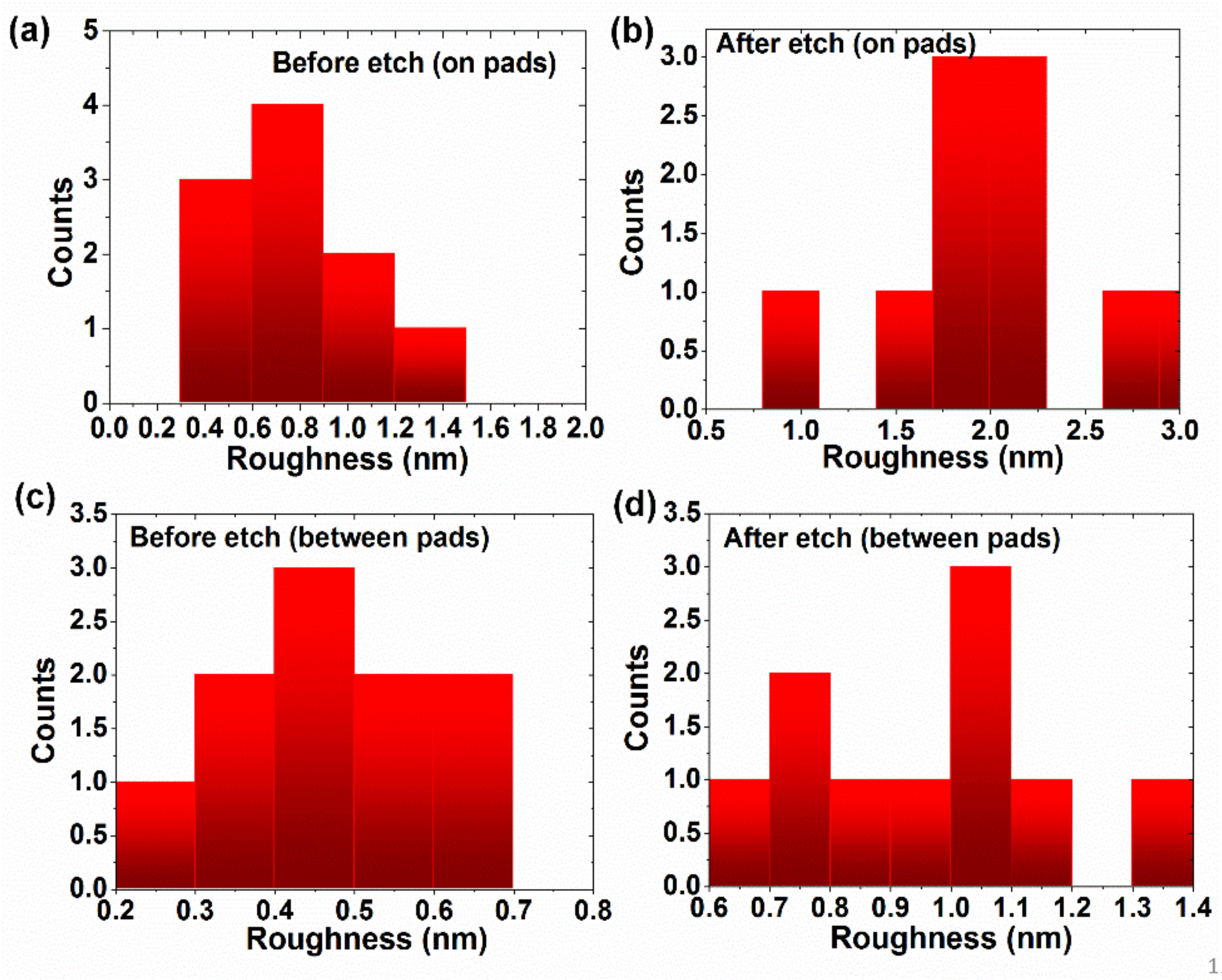

Supplementary figure 6. Comparison of the oxide roughness before and after planarization. (a) and **(b)** show the histograms of roughness of oxide measured on top the device pads ('red' and 'blue' pins) before and after the etch-back process respectively. **(c)** and **(d)** show the histograms of the oxide roughness measured between the device pads before and after the etch-back process respectively. In all the cases a slight increase is observed in the oxide roughness after the etch-back process.

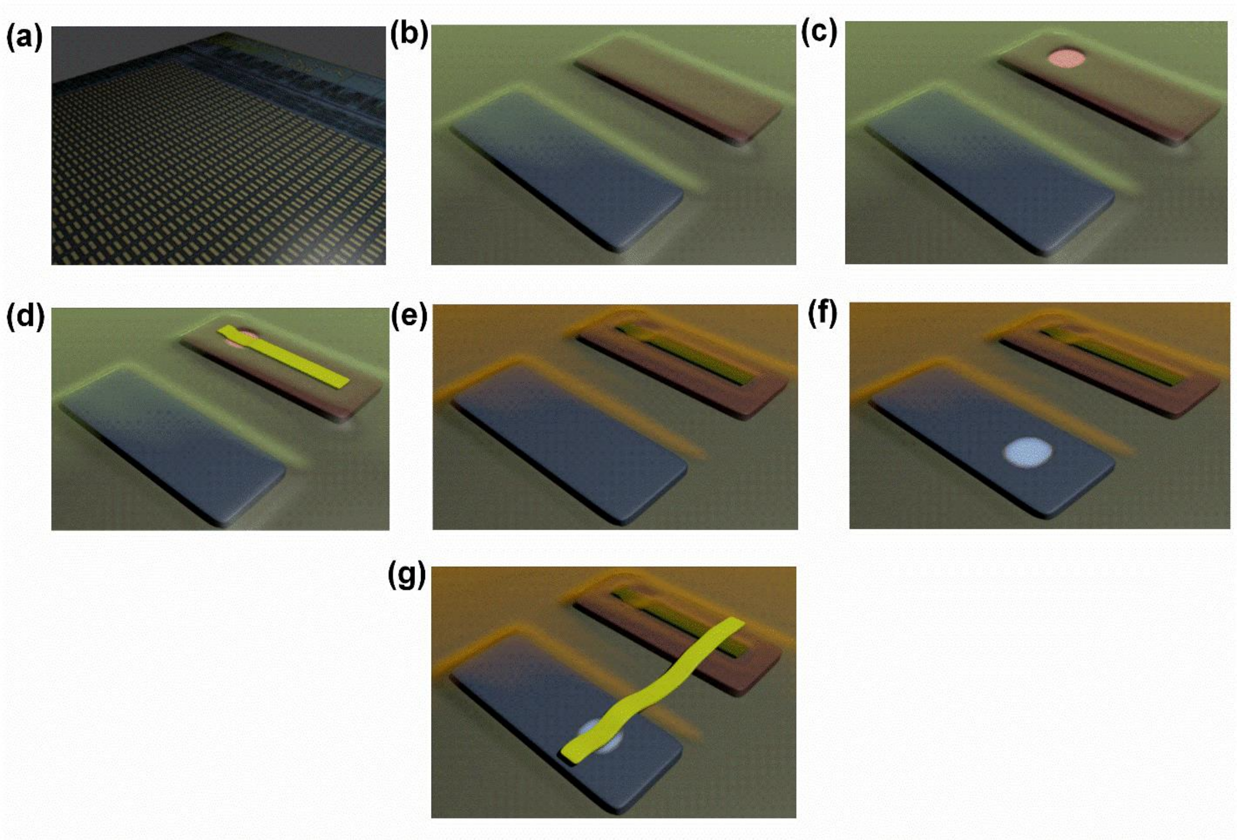

Supplementary figure 7. Device fabrication steps. (a) 3D image of the CMOS chip covered under the planarized and etched back oxide layer , **(b)** a pair of the 'Red' and 'Blue' pins corresponding to one 'CMOS cell' covered under the planarization oxide, **(c)** via opening on the 'red' pin by photolithography and dry etching for creating bottom electrode contact, **(d)** bottom electrode (Ta/Pt) deposition by photolithography and e-beam evaporation, **(e)** deposition of the switching dielectric (Al_2O_3/TiO_{2-x}) by reactive sputtering, **(f)** via opening on the 'blue' pin for creating top electrode contact, **(g)** deposition of top electrode (Ti/Pt) by photolithography and e-beam evaporation.

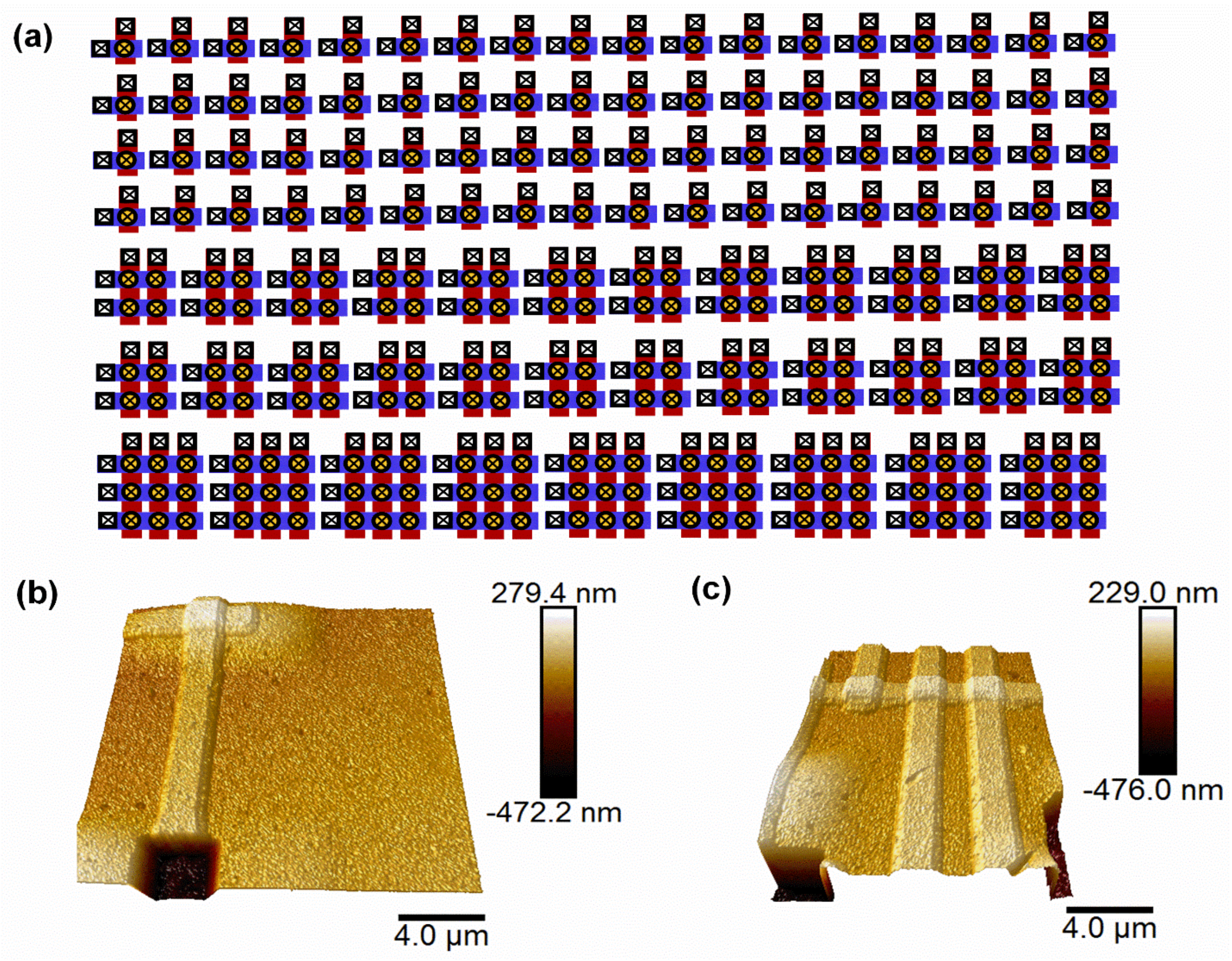

Supplementary figure 8. Details of the 2D single devices and crossbars. (a) Layout of the single devices as well as 2x2 and 3x3 crossbars (2 dimensional) integrated on the CMOS chip, **(b)** AFM image of a typical single cross-point device integrated, **(c)** section of a 2D crossbar integrated on the CMOS chip.

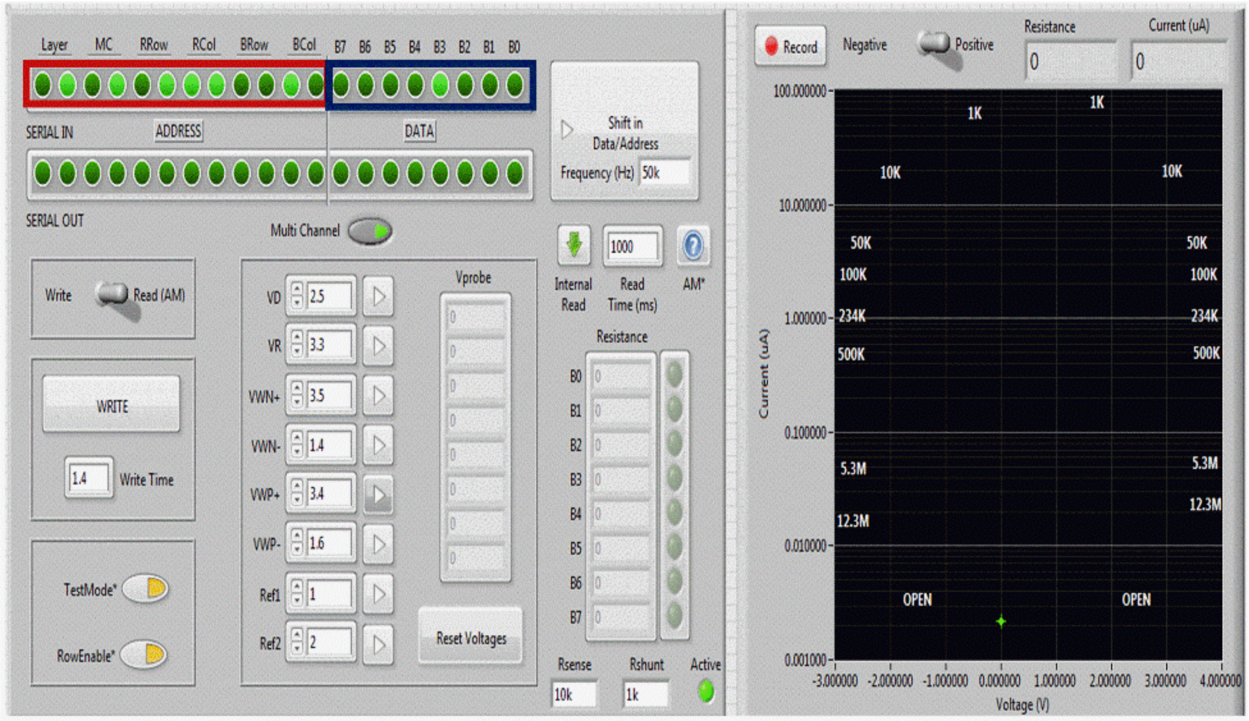

Supplementary figure 9. An image showing the interface to address each device integrated on a CMOS chip. The 12 bit address shown within the red box can select a cross-point within one multi-cell in the CMOS cell array. The 8 bits shown in the blue box can select 8 multi-cell columns concurrently.

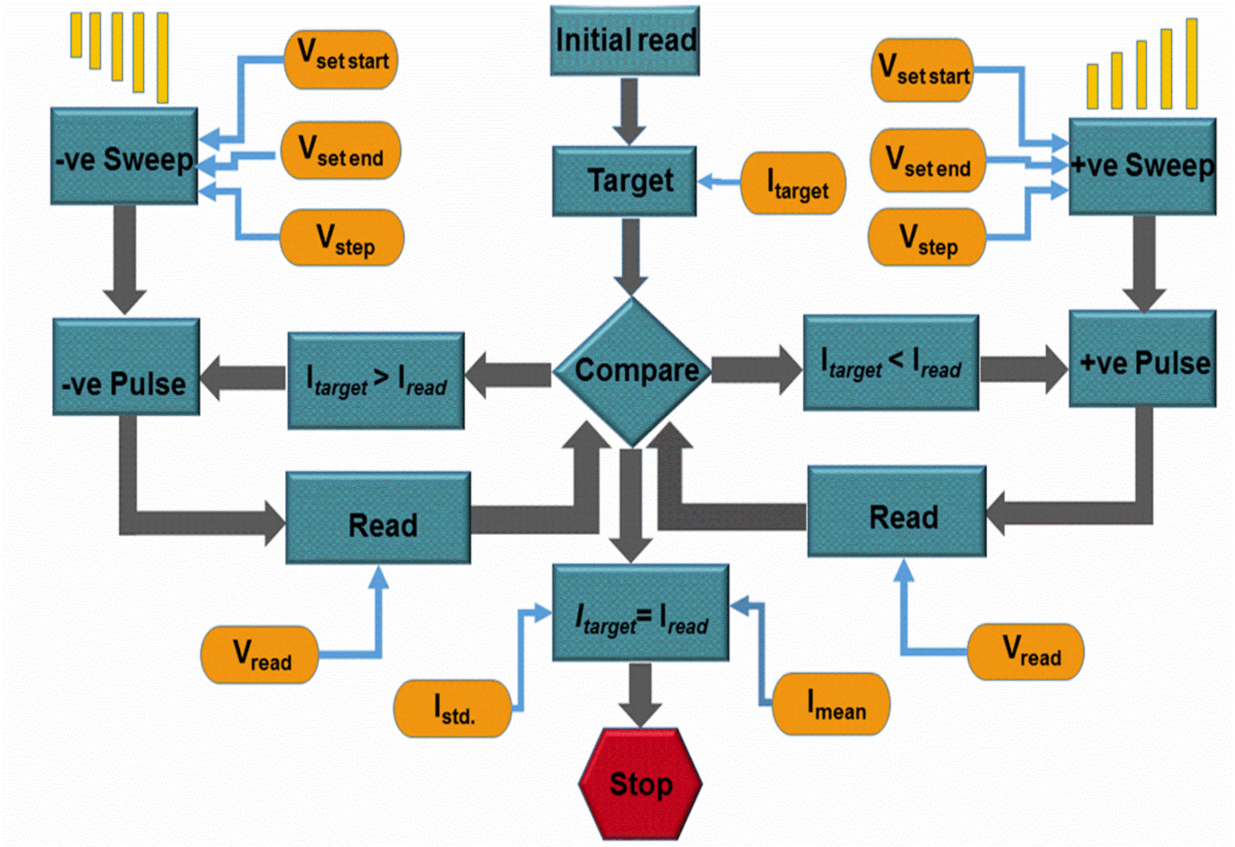

Supplementary figure 10. A flowchart showing the tuning algorithm to tune the integrated memristive devices. Orange boxes denote the user-input variables. At each point of the tuning the read current Iread is compared with the target current Itarget. The tuning operation stops at any point if Iread = Itarget. The accuracy of the match is governed by Imean and Istd. The tuning stops if (Target current – mean read current) ≤ Imean and the standard deviation of the read current matches Istd.

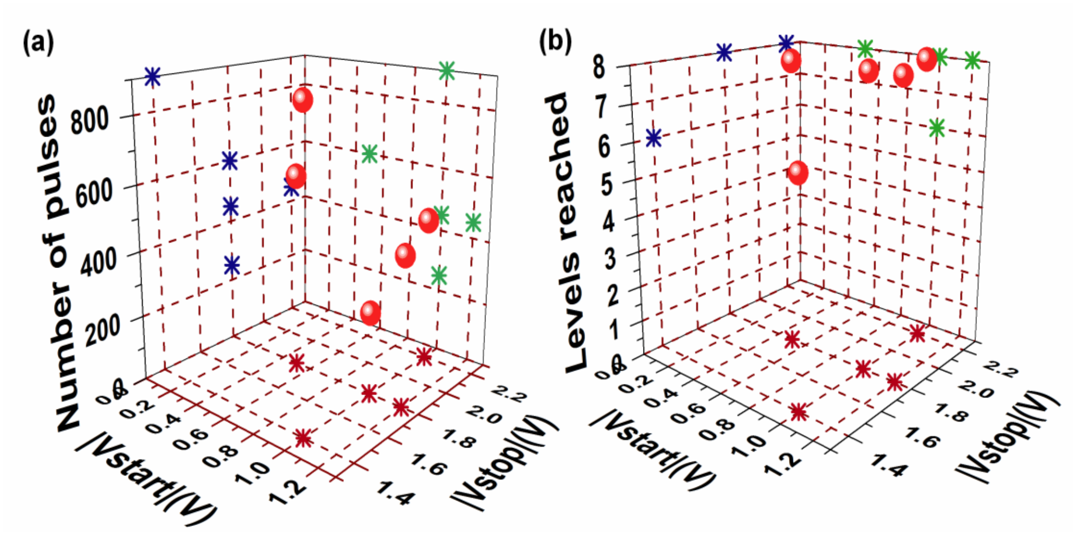

Supplementary figure 11. (a) optimization of pulse numbers as a function of Vstart and Vstop, **(b)** Number of levels reached for different Vstart and Vstop values.

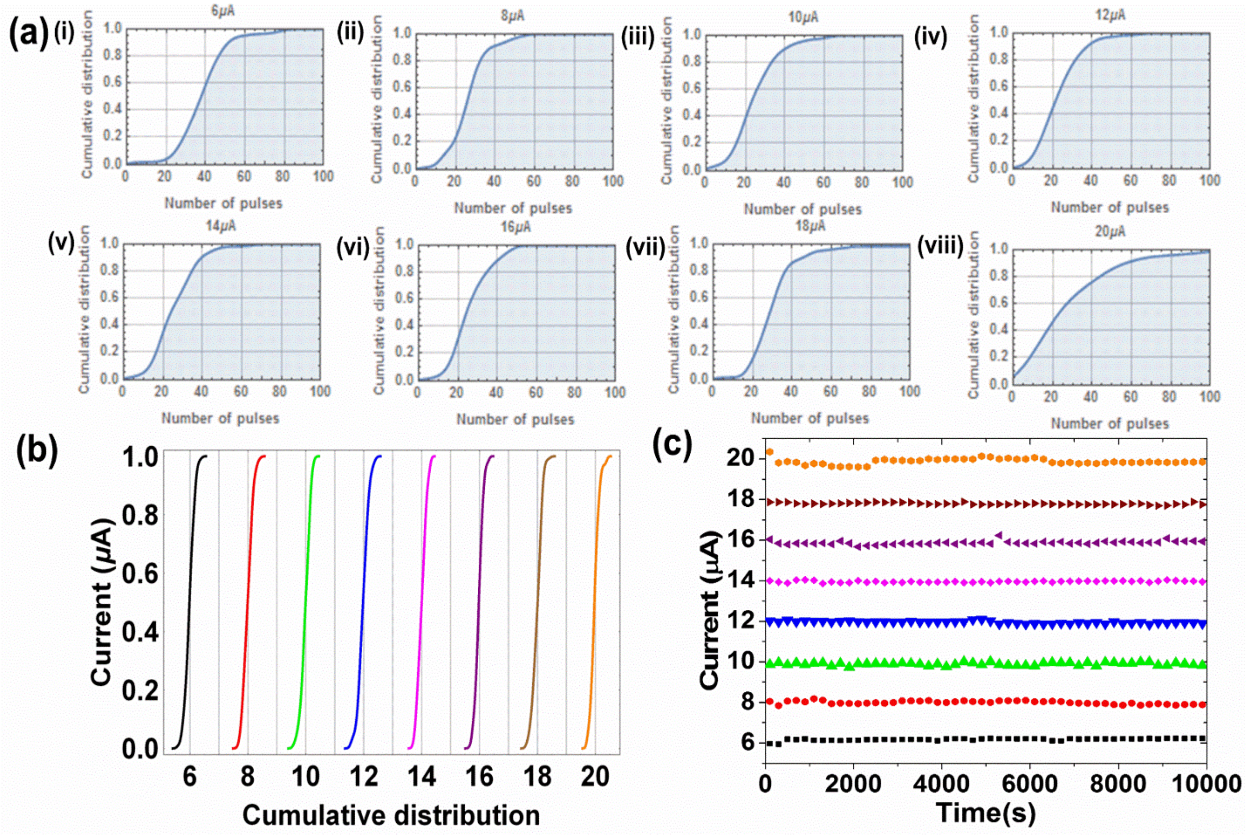

Supplementary figure 12. Performance of the optimized tuning algorithm for 1000 switching events randomly occurring between the 8 designated levels. (a) (i)-(a) (viii) depict the

cumulative distribution of the number of pulses required to tune to each of the eight levels, **(b)** cumulative distributions of the tuned current values for each level, **(c)** room temperature retention of each level measured over 10000 seconds.

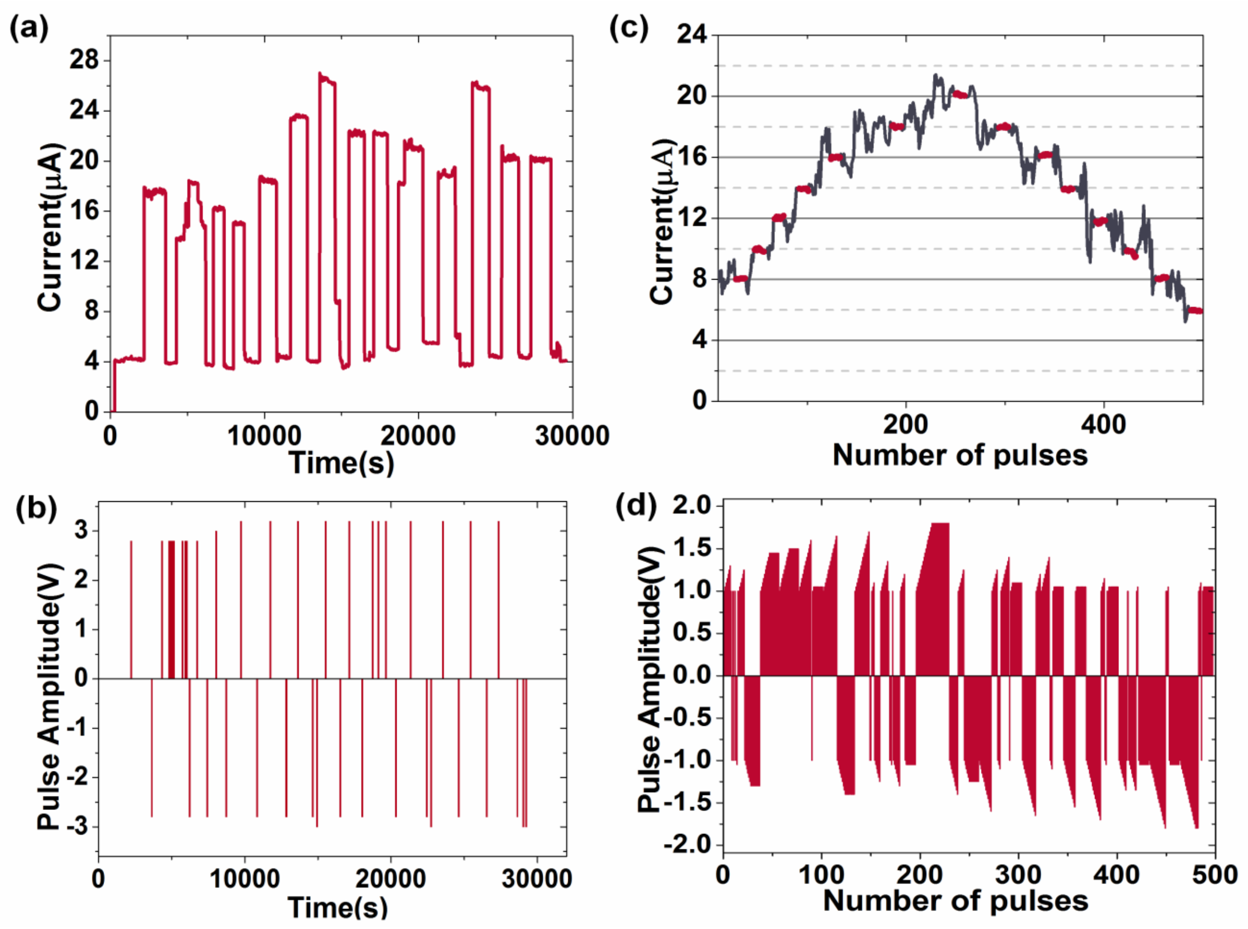

Supplementary figure 13. (a) Pulsed switching of a device between fully on and off states. **(b)** The applied write pulses corresponding to the switching shown in (a). **(c)** Example of the tuning operation using the train of positive and negative pulses shown in **(d)**. Note that the amplitudes of the pulses applied for the tuning procedure are significantly less compared to the pulse amplitude required for turning the device fully on or off.

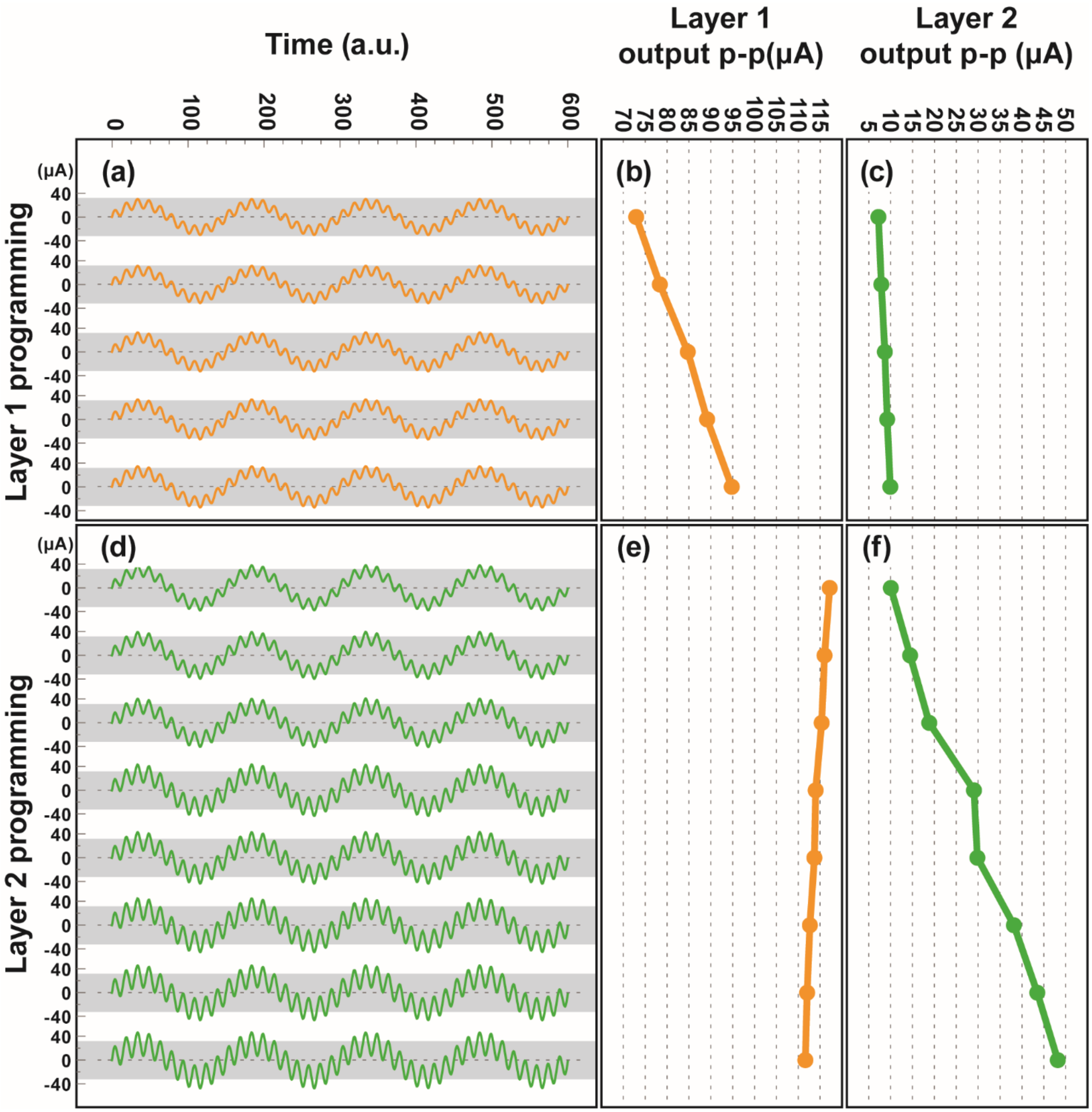

Supplementary figure 14. (a) Evolution of the output waveform as the conductance of the device in layer 1 is increased while the conductance of layer 2 device is kept unchanged. **(b)** Evolution of the component of the output current in layer 1 device as its conductance increases. **(c)** Component of the output current in layer 2 device remains unchanged. **(d)** Evolution of the output waveform as the conductance of the device in layer 2 is increased while the conductance of the layer 1 device is not changed. **(e)** Component of the output waveform in layer 1 corresponding to the experiment shown in (d). **(f)** Evolution of the component of output current in layer 2 as its conductance increases.

Supplementary Note 1. Tuning procedure

In each tuning step the read-current (Iread) measured at read-voltage Vread is compared against the target current (Itarget) corresponding to the desired state of the device. Depending on whether Iread is less or greater than the Itarget, a set of positive or negative pulses (1 µs) are applied in a ramp. Each write pulse is followed by a read operation (with a 1 ms read pulse) that evaluates the Iread. The tuning operation stops at any point if Iread matches Itarget. The precision of the tuning operation is governed by the user-defined inputs Imean and Istd. In case of an overshoot, the tuning operation can go in the opposite direction. All the user-defined inputs can be used to optimize the speed of the tuning operation.
